# Mapping of Functional Metabolic Phenotypes in Acute Myeloid Leukemia

**DOI:** 10.1002/cam4.70950

**Published:** 2025-05-19

**Authors:** Sissel Dyrstad, Kimberley Joanne Hatfield, Endre Stigen, Marte Karen Brattås, Karl Johan Tronstad, Håkon Reikvam

**Affiliations:** ^1^ Department of Biomedicine, Faculty of Medicine University of Bergen Bergen Norway; ^2^ Department of Immunology and Transfusion Medicine Haukeland University Hospital Bergen Norway; ^3^ K.G. Jebsen Center of Myeloid Malignancies, Institute of Clinical Science, Faculty of Medicine University of Bergen Bergen Norway; ^4^ Department of Medicine Haukeland University Hospital Bergen Norway

**Keywords:** acute myeloid leukemias, glycolysis, metabolic phenotypes, metabolism

## Abstract

**Background:**

Acute myeloid leukemia (AML) is an aggressive hematologic malignancy with a poor prognosis, particularly in older patients. AML is highly heterogeneous, influenced by various chromosomal, genetic, and epigenetic alterations.

**Methods:**

This study investigated the metabolic profiles of primary AML cells from 46 patients, focusing on mitochondrial respiration and glycolysis. We hypothesized that the metabolic profiles would reflect distinct disease characteristics. Using Seahorse technology, we measured the oxygen consumption rate (OCR) for mitochondrial respiration and the extracellular acidification rate (ECAR) for glycolysis.

**Results:**

Our results showed significant variability in metabolic activity, with some samples relying more on glycolysis than mitochondrial respiration. Mature AML cells (FAB M4/M5, CD34 negative) exhibited increased rates of both mitochondrial respiration and glycolysis, indicating distinct metabolic adaptations. Higher glycolytic activity was observed in patients with adverse cytogenetic abnormalities. However, no clear associations were found between metabolic profiles and mutations in *FLT3* or *NPM1*.

**Conclusion:**

These findings highlight the role of metabolic variability in AML and suggest that targeting specific metabolic pathways could offer therapeutic opportunities, particularly for subgroups like FAB M4/M5 with unique metabolic features. Further studies are needed to refine these therapeutic strategies for clinical application.

## Graphical abstract

Analysis of primary AML cells from 46 patients shows significant variability in metabolic activity, highlighting diverse metabolic phenotypes within the disease. AML cells classified as FAB M4/M5 exhibited higher metabolic rates compared to less mature phenotypes. The findings underscore the potential for targeted therapeutic strategies based on specific metabolic profiles.

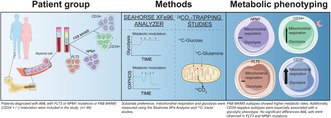

## 1 Introduction

Acute myeloid leukemia (AML) is a highly aggressive malignancy characterized by the rapid proliferation of immature myeloid leukemic blasts [1]. The uncontrolled accumulation of these malignant cells disrupts normal hematopoiesis and bone marrow function, resulting in severe bone marrow failure [1]. The disease is associated with high degrees of morbidity and mortality. Despite advancements in treatment approaches over the past decade, the prognosis remains poor, particularly for older patients > 65 years, with very few surviving the disease [2].

AML manifests as a heterogeneous disease, encompassing various chromosomal, molecular genetics, epigenetic, and biochemical alterations, all of which ultimately influence protein function and cell signaling pathways [3]. Recent advancements have shed light on the intricate biology of leukemic cells, allowing for the identification of distinct subgroups within AML patients. A prevalent subgroup is characterized by mutations in signaling genes, notably activating mutations of tyrosine kinases (TKs) such as Fms‐like tyrosine kinase 3 (*FLT3*), found in approximately 30%–40% of AML cases [4]. This mutation has generally been associated with high relapse and dismal prognosis, although improvement in treatment outcomes has occurred in the last decade [4]. On the other hand, we find the nucleophosmin 1 (*NPM1*) mutation, which is present in approximately 40% of AML patients and has traditionally been associated with a favorable prognosis, particularly in the absence of *FLT3* mutation [5].

Emerging evidence suggests that malignant transformation, including in AML, profoundly impacts cellular energy metabolism [6, 7]. Varied metabolic phenotypes have been proposed across different AML subgroups, hinting at diverse utilization of metabolic pathways by distinct driver mutations [8], although this relationship is not yet firmly established. Cancer cells seem to require glutamine for increased biosynthetic activity, with its conversion to glutamate by glutaminase (GLS) being a critical step. It has been demonstrated that glutamine levels control mitochondrial oxidative phosphorylation (OXPHOS), with the GLS isoform glutaminase C (GLSC) being the most abundantly expressed in AML cells [9]. Inhibiting GLS1, either by knockdown or with the drug CB‐839, reduces OXPHOS, arrests leukemic cell proliferation, and induces apoptosis without affecting normal progenitors, significantly inhibiting AML development in mice [9]. Hence, targeting metabolic pathways has emerged as a promising strategy in the pharmacological management of malignant diseases, with the metabolism of leukemic cells potentially serving as their “Achilles' heel” [6].

In the present study, we hypothesized that the metabolic profile of primary AML cells depends on the leukemic molecular phenotype and genotype and therefore demonstrates significant heterogeneity. By thorough live‐cell analysis of key metabolic functions, we aimed to identify new discriminatory characteristics among AML patients based on their metabolic signatures.

## 2 Material and Methods

### 2.1 Patients and Primary Human AML Cells

The collection and use of samples for this study were approved by the Regional committees for medical and health research ethics (REK), both for biobanking and in vitro experimental research (REK 1750/2015 and REK 480847/2022). Registration of collected samples is also approved by the Norwegian Data Protection Authority (reference 02/1118‐5). Peripheral blood mononuclear cells were obtained from AML patients at diagnosis after written informed consent was obtained. AML cells were isolated by density gradient separation (Lymphoprep, Alere Technologies, Norway) and contained at least 95% leukemic blasts before being cryopreserved in RPMI‐1640 (Sigma‐Aldrich, St. Louis, MO, USA) with 10% dimethyl sulfoxide (DMSO) (Merck KGaA, Gernsheim, Germany) and 20% heat‐inactivated fetal bovine serum (FBS) (BioWest, Nuaille, France). All samples were stored in liquid nitrogen until thawed and used in experiments. In our study, cells were derived from 46 consecutive AML patients, and their main characteristics are given in Table 1.

**TABLE 1:** Patient characteristics. Demographical and clinical characteristics of the 46 AML patients included in the study.

Patient characteristics		observations
Demographic data and disease history		
Gender (number, percent)	Female/male	22/24 (48/52)
Age (median, range)	Years	60 (19–95)
Disease history (number, percent)	De novo	39 (85)
	Secondary	7 (15)
Hematology (mean, range)		
Hemoglobin, g/dL		9.9 (6.1–14.0)
Platelets, ×10^9^/L		52 (8–632)
WBC, ×10^9^/L		58.0 (4.9–241)
AML cell differentiation (number, percent)		
FAB	M0‐1	10 (22)
	M2	12 (26)
	M4‐5	22 (48)
	Unclassified	2 (4)
CD34	Positive (≥ 30%)	24 (52)
	Negative (≤ 30%)	18 (39)
	Unclassified	4 (9)
Cytogenetic and genetic abnormalities (number, percent)		
Cytogenetics^a^	Favorable	4 (9)
	Intermediate	33 (72)
	Adverse	9 (20)
*FLT3*	Wild type	32 (70)
	ITD	14 (30)
	TKD^b^	1 (2)
*NPM1*	Wild type	27 (59)
	Insertion	17 (37)
	Unknown	2 (4)

*Note:* Favorable include: Inv (16) (2) and t(8;21) (2). Intermediate include normal karyotype (26), del7q (1), del9 (1), t(1;13) (1), trisomy 8 (2), trisomy 13 (1), del20 (1). Adverse include: t(8;16) (1), Inv(t(3;3) (2), t(v;11) (2), Del7 (1), Complex (3).

Abbreviations: FAB, French‐American‐British; *FLT3*, FMS‐like tyrosine kinase 3; ITD, internal tandem duplication; *NPM1*, nucleophosmin 1; TKD, tyrosine kinase domain; WBC, white blood cell count.

^a^ Cytogenetic risk classification is based on the European Leukemia Net Classification of 2022.

^b^ One patient harbored both ITD and TKD mutation.

### 2.2 Rates of Mitochondrial Respiration and Glycolysis

Oxygen consumption rate (OCR) and extracellular acidification rate (ECAR) were measured using the Seahorse XFe96 instrument (Agilent, Santa Clara, CA, USA). All compounds were from Sigma‐Aldrich unless otherwise stated. To mimic the native situation in circulating blood, we used a human plasma‐like medium (HPLM, Gibco, Thermo Fisher Scientific, Waltham, MA, USA) as assay medium. However, we used a custom‐made version produced without glutamine, riboflavin, bicarbonate, glucose, and pyruvate, which we supplemented with 5 mM HEPES, 5.5 mM glucose, 2 mM pyruvate, and 2 mM glutamine, and adjusted pH to 7.4. For the extracellular flux analysis, the concentrations of compounds, the cell number per well, the careful handling of cells, and the execution of measurement were all optimized through preparatory experiments. The standardized AML cell metabolism protocol involved the sequential addition of oligomycin (2 μM), carbonyl cyanide m‐chlorophenylhydrazone (CCCP, 2 μM) and rotenone (1 μM)/antimycin A (1 μM). Preparatory investigations showed significant variation in metabolic rates between samples. The time expenditure of the measurement protocol was minimized to obtain the best possible reflection of the endogenous rates.

The cells were analyzed immediately after gentle thawing from their cryopreserved state. The ampules were thawed in a 37°C water bath and diluted with 3 mL HPLM assay medium before the viable cell number was determined using trypan blue exclusion. The cell suspension was centrifuged at 290× *g* for 5 min, and the supernatant was replaced with fresh HPLM assay medium, using a volume giving 1.5 million viable cells/mL. An amount of 80 μL of the cell suspension, that is, 120,000 viable cells, was transferred to each well of the special 96‐well cell culture plate. An optimized centrifugation‐aided protocol was used to facilitate a homogenous distribution of the cells in the wells, which were coated with 3 μg/cm2 CellTak according to the manufacturer's instructions (Corning, Steuben County, NY, USA). After adding the medium and cells, the plate was centrifuged in an Eppendorf 5810 centrifuge at an acceleration rate of 4 with the break off. The first centrifugation was stopped when it reached 41× *g*. The orientation of the plate was then changed by 180°, and the centrifugation was repeated and stopped at 85× *g*. The cells were inspected by microscopy to control that they were evenly spread in the wells, and the plate was incubated for 25–30 min at 37°C without CO_2_. Immediately before measurement was started, 95 μL of pre‐warmed HPLM assay medium was carefully added to each well and the plate was inserted into the Seahorse instrument.

Thawing and pre‐incubation of the cells, and calibration of the instrument, took maximum of 3 h before the start of the analysis. The analytical protocol included 12 min equilibration, three cycles of 3 min mix and 3 min measurement at baseline, one cycle of 3 min mix and measurement after injection A‐C, and two cycles of 3 min mix and measurement after D, giving a total protocol time of approximately 60 min (excluding calibration of the utility plate). On each analytical plate, HL60 cells (80,000 cells/well) thawed and treated in the same manner as the patient AML cells were included to enable plate‐internal quality assessment of the experiment.

### 2.3 Oxidation Rates of Glucose or Glutamine to CO_2_

In parallel with the analysis of mitochondrial respiration and glycolysis, 200,000 cells/well were plated for investigating oxidation rates by C^14^‐labeled substrates as described previously [10, 11], with some modifications. The assay medium consisted of HPLM medium containing 5 mM HEPES (pH 7.4). The labeled substrates used were [U‐14C] glucose (4 μCi/mL) and [U‐14C] glutamine (1 μCi/mL) from PerkinElmer (Waltham, MA, USA). Respective amounts of non‐labeled substrates were added to obtain standard final concentrations of 5.5 mM glucose and 2 mM glutamine in the assay medium. HL60 cells (80,000 cells/well) were included on each plate as an experimental control. The ^14^CO_2_ produced by the cells was trapped [10, 11] using an incubation time of 2 h (standard incubator, 37°C, 5% CO_2_).

### 2.4 AML Cell Proliferation Assay

AML cells were seeded in triplicates at 50,000 cells/well in 100 μL of StemSpan SFEM supplemented with G‐CSF, SCF, and FLT3 ligand in flat‐bottomed 96‐well plates. The cultures were incubated at 37°C in a humidified atmosphere for 6 days before 20 μL [^3^H]‐thymidine (TRA 310; Perkin Elmer, NET027A005MC, Amersham International, Amersham, UK) was added. After an additional day, cultures were harvested, and nuclear incorporation was measured using the [^3^H]‐thymidine incorporation assay.

### 2.5 Statistical and Bioinformatical Analysis

The measured extracellular flux rates (OCR, ECAR) and CO_2_‐trapping rates were normalized to viable cell count per well. Mean viability of the samples with detectable metabolic activity was 92% (range 78%–99%). For each sample, 4–8 replicate wells were measured on the same plate. The data from each well and sample was carefully evaluated based on systemic technical performance (calibration, background, edge effects) and experimental quality (detection limit, modulator responses, stability). Accordingly, six samples that showed very low extracellular flux rates and no response to chemical modulators (Seahorse instrument) were regarded as non‐viable under these conditions and were not included in the statistical analysis. For each output, means were calculated based on intra‐plate replicates. For samples that were successfully measured twice (16 samples), the inter‐plate means were calculated subsequently. Both the measurements and data analyses were performed blinded in terms of patient characteristics and AML blast classification, that is, this information was not available for the persons involved. The relationships with molecular and genetic AML subsets were studied subsequently. Generative artificial intelligence (AI) was sometimes used in the writing process to translate words or improve readability in certain sections.

## 3 Results

### 3.1 Diverse Metabolic Phenotypes Were Observed in Primary AML Cells

**FIGURE 1:**
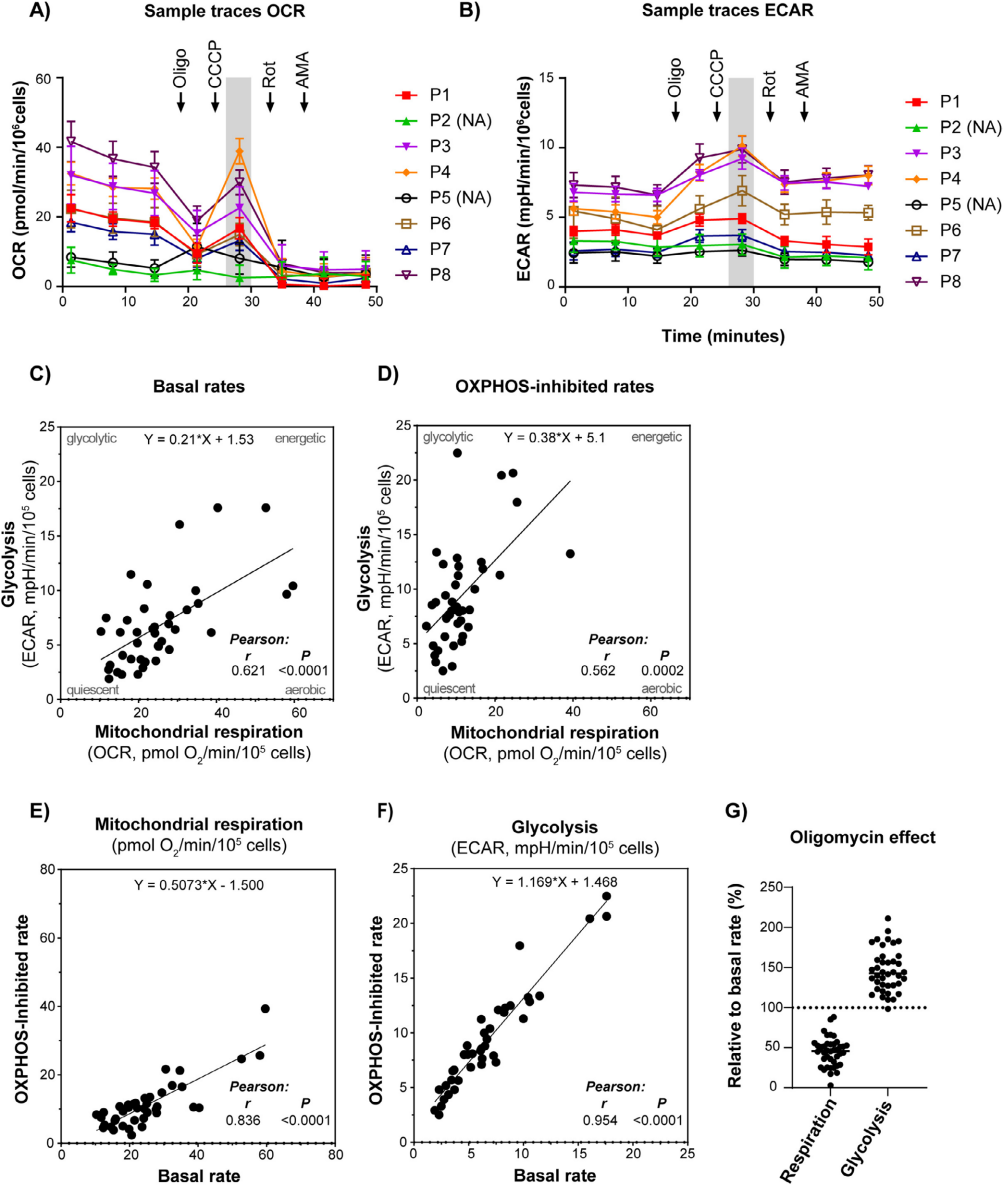
Energy metabolic function in primary AML cells. Mitochondrial respiration (oxygen consumption rate, OCR, pmol O_2_/min/10^5^ cells) and glycolysis (extracellular acidification rate, ECAR, mpH/min/10^5^ cells) were measured in primary AML cells from 46 patients. (A) and (B) Representative traces showing data for one analysis plate with 8 samples (randomly numbered; NA, no activity). Using a pre‐optimized protocol, oligomycin (oligo), CCCP, rotenone (Rot), and antimycin A (AMA) were added successively to modulate metabolic parameters. The basal and OXPHOS‐inhibited rates, that is, the rates before and after oligomycin addition, respectively, were analyzed statistically. Since a significant proportion of samples did not tolerate well the addition of CCCP, the resulting uncoupled respiration rate was excluded as descriptor parameter (as indicated on the figure). (C) The association between basal rates of mitochondrial respiration and glycolysis was used to study differences in cellular metabolic phenotype. (D) In the OXPHOS‐inhibited state after adding oligomycin, there was an overall shift towards a glycolytic phenotype. (E) and (F) display the relationships between the rates of mitochondrial respiration and glycolysis, respectively, in the absence (basal rate) and presence (OXPHOS‐inhibited rate) of oligomycin. (G) The relative change (%) caused by oligomycin (OXPHOS‐inhibition). All data are shown as the mean of 4–8 technical replicates for each subject, or the averaged mean if repeated experiments were performed (for 16 subjects). Pearson correlation analysis and simple linear regression analysis were used in (C–F).

We examined the rates of mitochondrial respiration, that is, OCR, and glycolysis, that is, ECAR, in cultures of native AML cells collected from 46 patients (Table 1 and Supporting Information), using a pre‐optimized extracellular flux analysis protocol. Metabolic activity was detectable in 40 of the 46 samples analyzed (87%). Data from a representative run with a panel of 8 patients are shown in Figure 1A,B. The rate of uncoupled mitochondrial respiration measured after the addition of the uncoupler, CCCP, was lower than the basal rate for several samples. This indicates that the cells were not able to uphold the resulting high uncoupled rates, possibly due to depletion of key metabolites and loss of mitochondrial integrity. Such an effect when using CCCP is not uncommon when measuring under these conditions (intact cells, cell culture medium). Since we were not able to pursue this further due to the limited sample amount, we decided to exclude the CCCP‐uncoupled respiration rate as a descriptor parameter (as indicated by the shaded areas in Figure 1A,B), and rather focused on the basal rate and the effect of the ATP synthase inhibitor, oligomycin. The OXPHOS‐inhibited metabolic state caused by oligomycin resembles a classic hypoxia‐induced metabolic shift, and we employed this as an approach to evaluate the functional metabolic phenotype in AML samples from the patients.

The data revealed significant heterogeneity between samples in basal and OXPHOS‐inhibited (with oligomycin) rates of mitochondrial respiration (OCR, pmol O_2_/min/10^5^ cells; mean 25.2; range 10.4–59.5) and glycolysis (ECAR, mPH/min/10^5^ cells; mean 6.7; range 1.9–17.6) (Figure 1 and Supporting Information). Since high rates of mitochondrial respiration generally correlated with high rates of glycolysis, the heterogeneity between samples appears to largely reflect differences in total metabolic flux rates (Figure 1C,D). However, some samples exhibited a more glycolytic phenotype, that is, a high glycolytic rate relative to mitochondrial respiration (Figure 1C). Statistical analyses (not shown) revealed no relationship with age, sex, or hemoglobin level of the subjects. When oligomycin was added to the cells, there was a clear shift towards a more glycolytic phenotype, as expected (Figure 1D).

When comparing the overall rates in the absence versus presence of oligomycin, there was a significant reduction of mitochondrial respiration (average 25.2 vs. 11.3 pmol O_2_/min/10^5^ cells, *p* < 0.0001, *t*‐test) and a significant increase in glycolysis (average 6.7 vs. 9.3 mPH/min/10^5^ cells, *p* = 0.0091, *t*‐test). Although there was a relationship between the rates observed in the absence versus presence of oligomycin (Figure 1E,F), the relative effects differed significantly, ranging from 19% to 88% reduction of mitochondrial respiration and 0% to 109% increase of glycolysis (Figure 1G). Such differences in OXPHOS‐dependency may have significant implications related to bioenergetics, metabolic adaptations, cellular stress mechanisms, and proliferation.

### 3.2 AML Cell Metabolic Rates in Human Plasma‐Like Medium (HPLM) Compared to Conventional Culture Medium (DMEM) and Proliferation Status

**FIGURE 2:**
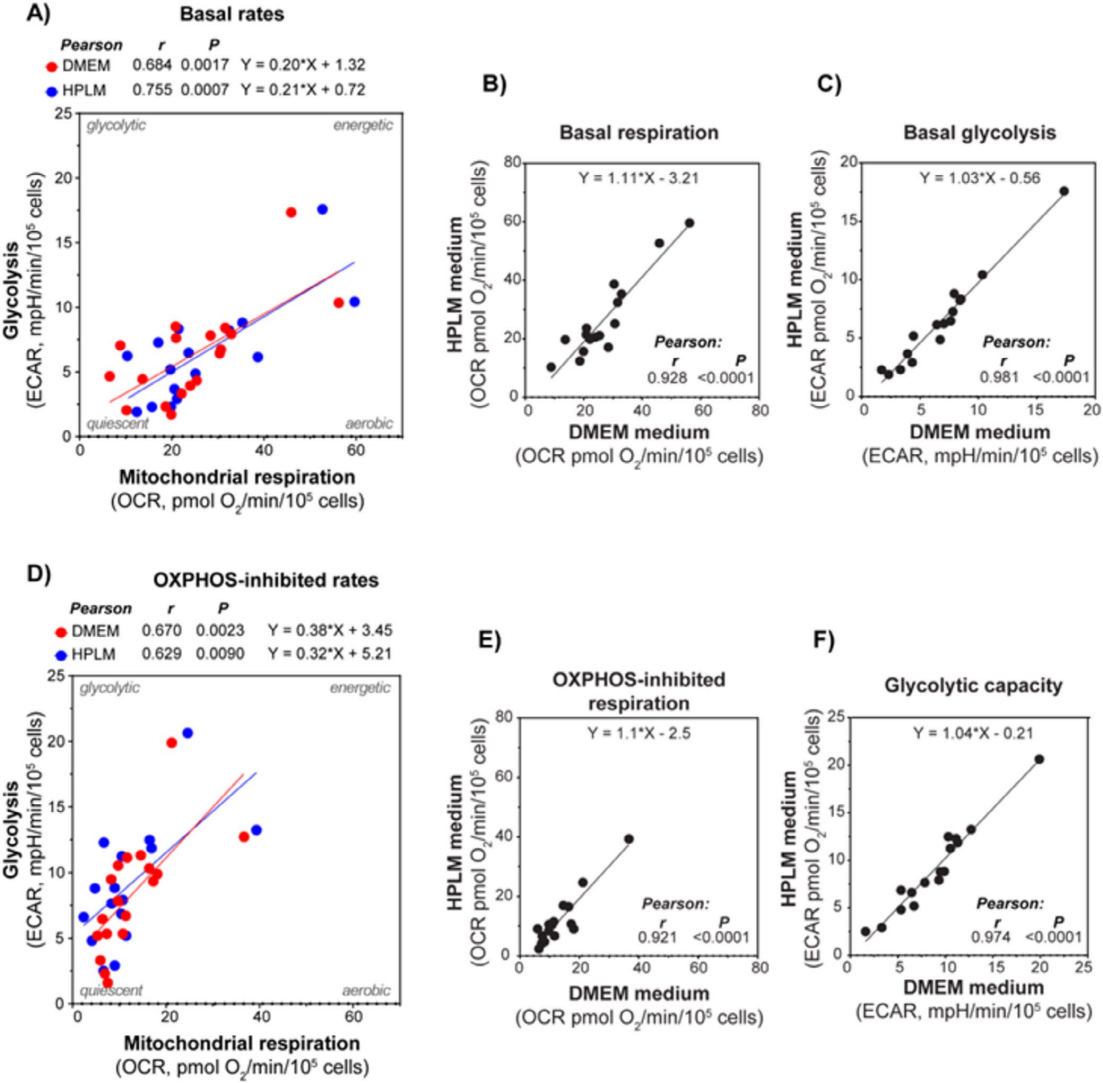
Comparison of cellular metabolic rates using HPLM and DMEM medium. The rates of mitochondrial respiration (oxygen consumption rate, OCR) and glycolysis (extracellular acidification rate, ECAR) when measured in DMEM and HPLM were compared using AML cells from 16 patients. (A) Correlation plot of the basal rates of mitochondrial respiration and glycolysis in the two media. (B) and (C) Correlation plots of DMEM versus HPLM for basal rates of mitochondrial respiration and glycolysis, respectively. (D–F) Show the OXPHOS‐inhibited condition (with oligomycin). All data are shown as mean of 4–8 technical replicates for each subject. Pearson correlation analysis and simple linear regression analysis were performed.

For a group of samples, we compared the metabolic rates obtained using HPLM versus DMEM as assay media in a side‐by‐side experimental setup (Figure 2). A strong correlation between the data obtained using the two different media was detected. This indicates that the cells can compensate for the possible impacts caused by the differences in the nutritional composition between the media. Hence, both media were found to provide a sufficient supply of energy substrates and other nutrients to maintain steady rates of the main energy pathways. We also compared whether metabolic rates were correlated to cytokine‐mediated proliferation of the leukemic blasts. Cytokine‐mediated proliferation was obtained by measuring 3H‐thymidine incorporation after 7 days, with samples from 22 patients. The proliferation rate was measured by scintillation counting, and median incorporation was 12,977 cpm, with a range of 12,112–58,680 cpm. We correlated both OCR, ECAR, and the OCR/ECAR ratio with proliferation rates, although no significant correlations were detected.

### 3.3 AML With Maturation (FAB M4/M5, CD34 Negative) Demonstrated Higher Rates of Mitochondrial Respiration and Glycolysis

**FIGURE 3:**
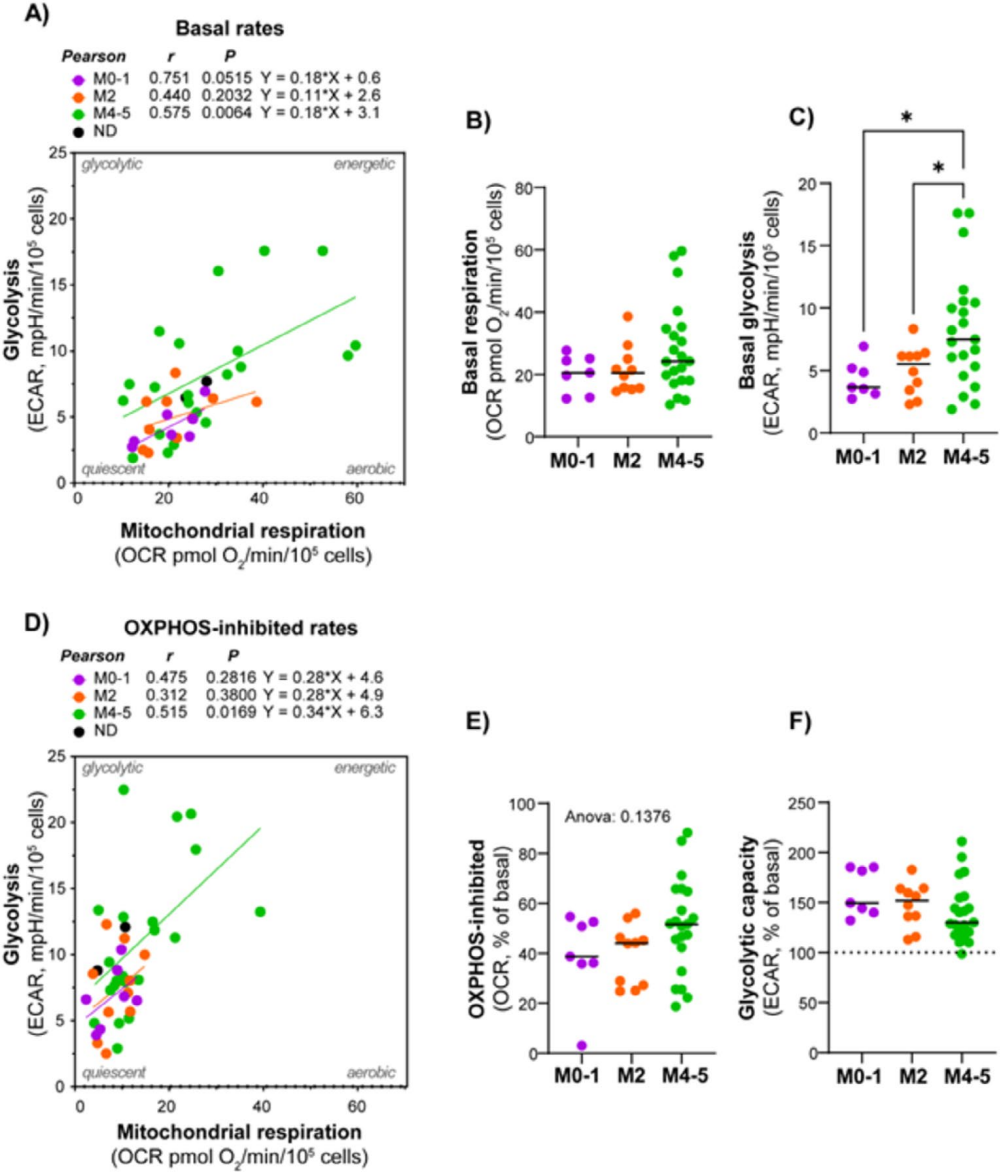
Relationship between the metabolic phenotype and AML maturation. Comparison of mitochondrial respiration (oxygen consumption rate, OCR) and glycolysis (extracellular acidification rate, ECAR) in AML maturation subsets. (A) Correlation analysis between the basal rates of mitochondrial respiration and glycolysis, comparing different maturation subsets according to the FAB classification. (B) Corresponding analysis under the OXPHOS‐inhibited condition. (C–F) Statistical analysis of key parameters according to FAB subsets. All data are shown as the mean of 4–8 technical replicates for each subject. Pearson correlation analysis and simple linear regression analysis were performed in (A) and (B). *p ≤ 0.05, ordinary one‐way ANOVA and uncorrected Fischer's LSD for multiple comparisons.

We aimed to investigate whether certain metabolic properties were associated with diagnostic and prognostic markers by comparing metabolic characteristics in subgroups showing different maturation based on FAB classification. The data showed a tendency of higher metabolic rates in the M4/M5 subclasses, compared to the M0/M1 and M2 subclasses. This was particularly evident and statistically significant for the glycolytic rate (Figure 3).

**FIGURE 4:**
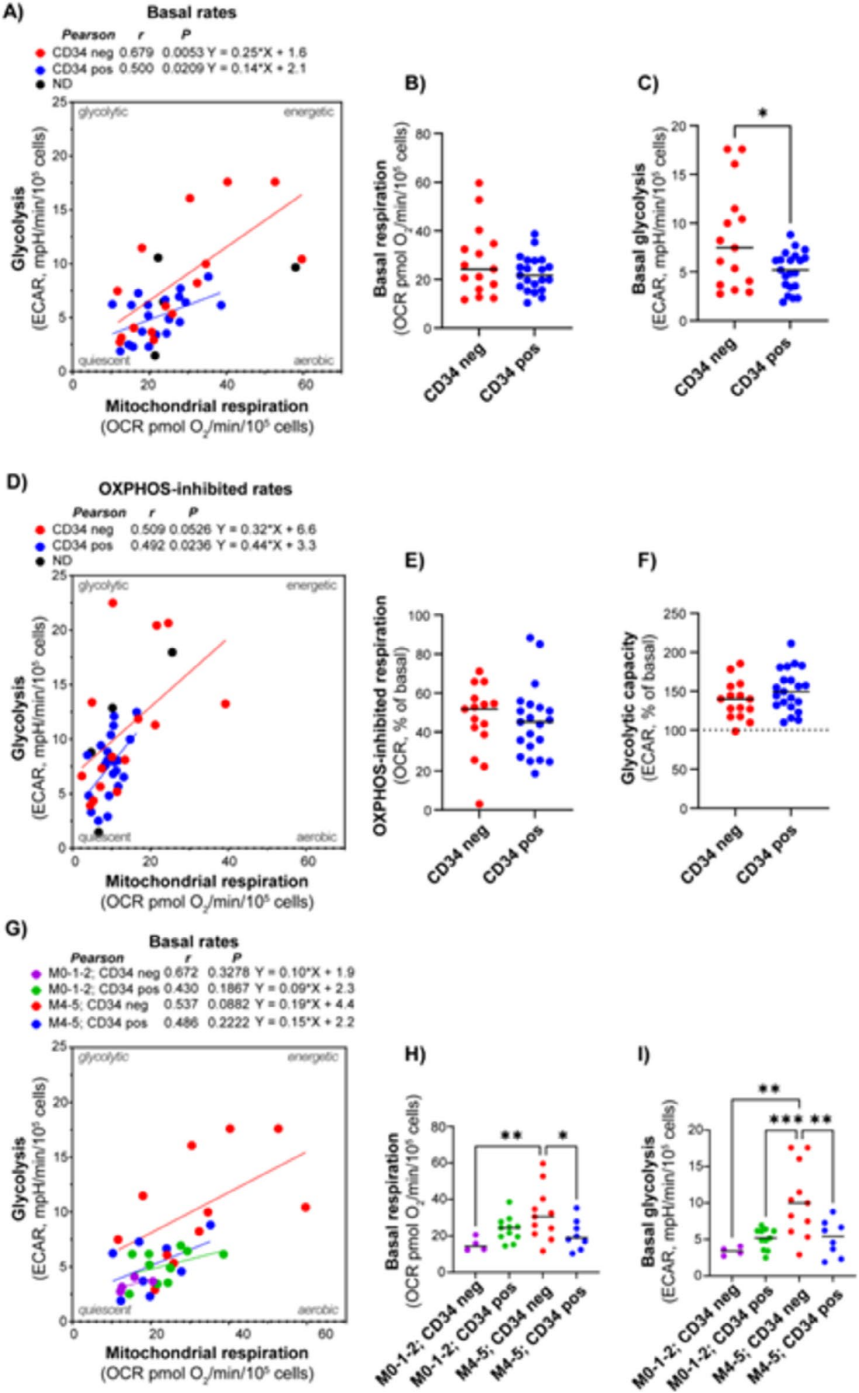
Relationship between the metabolic phenotype and CD34. Comparison of mitochondrial respiration (oxygen consumption rate, OCR) and glycolysis (extracellular acidification rate, ECAR) in CD34 positive versus CD34 negative subsets. (A) Correlation analysis between the basal rates of mitochondrial respiration and glycolysis, comparing CD34 positive and negative subjects. (B) Corresponding analysis under the OXPHOS‐inhibited condition. (C–F) Show statistical analysis of key parameters, according to CD34 status. (G–I) Corresponding analyses according to the combined FAB classification and CD34 status. All data are shown as mean of 4–8 technical replicates for each subject. Pearson correlation analysis and simple linear regression analysis were performed in (A), (B), and (G). *p ≤ 0.05, **p ≤ 0.01, ***p ≤ 0.001, *p ≤ 0.05 ordinary one‐way ANOVA and uncorrected Fischer's LSD for multiple comparisons.

When comparing CD34 positive and negative subjects, there was no difference in mitochondrial respiration; however, the CD34 negative group exhibited a slightly higher glycolytic rate (Figure 4A–F). Interestingly, when analyzed according to both FAB class and CD34 status, AML cells with both morphological and immunophenotypic signs of maturation (i.e., FAB M4/M5 and CD34 negative) demonstrated significantly higher rates of both mitochondrial respiration and glycolysis (Figure 4G–I). The AML subjects with the most prominent glycolytic phenotype belonged to this group. The proportion of mitochondrial respiration associated with ATP synthesis (i.e., the oligomycin‐inhibited proportion) was relatively similar between the AML subtypes. Despite the small sample size, the data suggest that AML cells with maturation have particularly high rates of energy metabolism. This may indicate higher energy demand compared to other subsets.

### 3.4 Adverse Cytogenetics Was Associated With Higher Glycolytic Rate, but Not Mitochondrial Respiration

**FIGURE 5:**
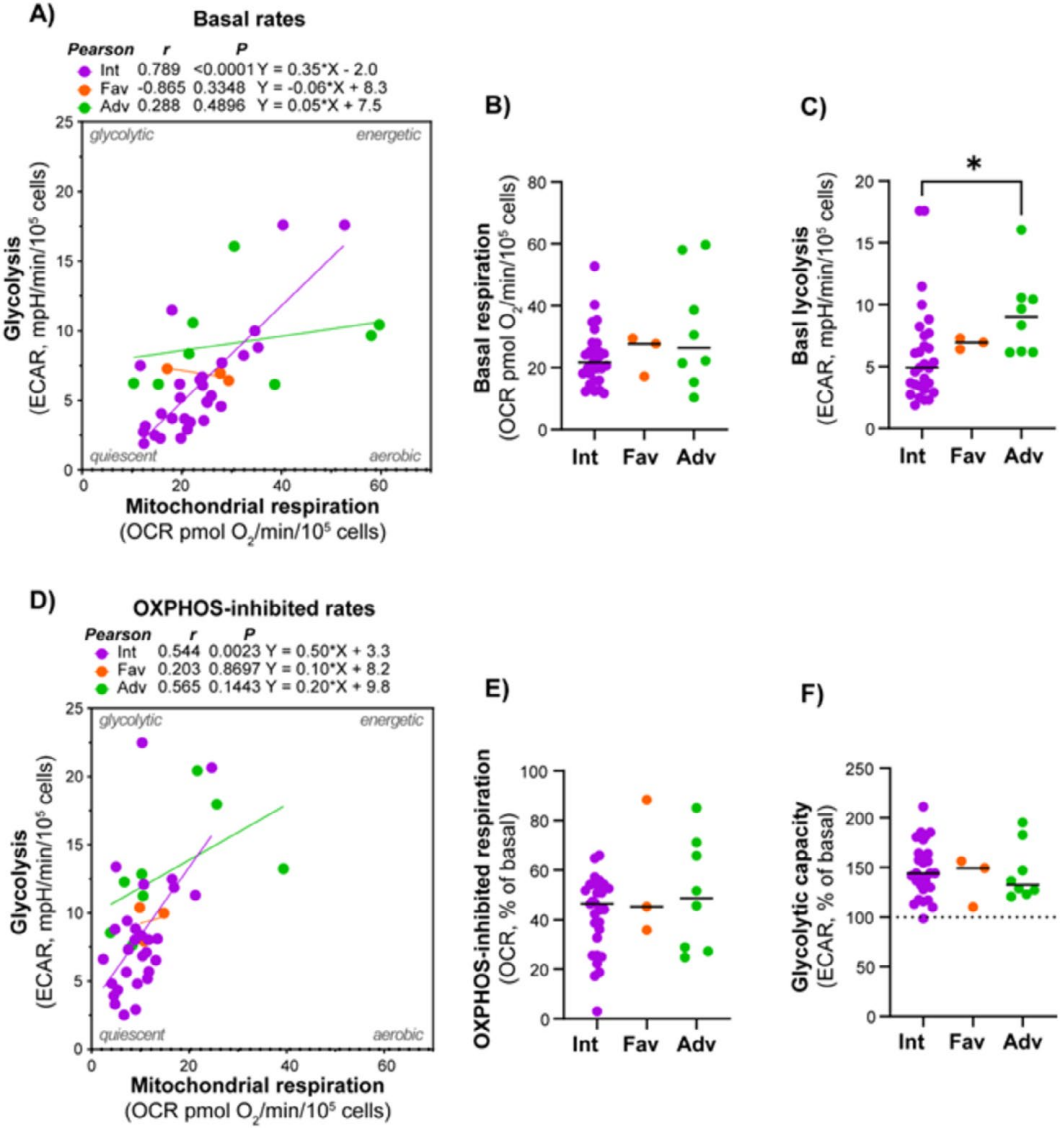
Relationship between the metabolic phenotype and adverse cytogenetics. Comparison of mitochondrial respiration (oxygen consumption rate, OCR) and glycolysis (extracellular acidification rate, ECAR) between different cytogenetic subsets. (A) Correlation analysis between the basal rates of mitochondrial respiration and glycolysis, comparing primary blasts from patients with favorable (Fav), intermediary (Int), and adverse (Adv) cytogenetics. (B) Corresponding analysis under the OXPHOS‐inhibited condition. (C–F) Show statistical analysis of key parameters, according to cytogenetic group. All data are shown as mean of 4–8 technical replicates for each subject. Pearson correlation analysis and simple linear regression analysis were performed in (A) and (B). *p ≤ 0.05, ordinary one‐way ANOVA and uncorrected Fischer's LSD for multiple comparisons.

Given the significant role of cytogenetics in treatment outcomes and prognosis in AML, we investigated the connection between metabolic and cytogenetic abnormalities. Patients were classified according to cytogenetic abnormalities using the ELN 2022 classification into good, intermediate, and poor prognosis groups [12]. There was a strong association between mitochondrial respiration and glycolytic rates in subjects with intermediate cytogenetics, but not in the favorable and adverse cytogenetics groups; however, these included significantly fewer subjects (Figure 5). The adverse cytogenetics group exhibited a significantly higher glycolytic rate compared to the intermediate group.

### 3.5 No Clear Associations Between Metabolic Profile and *FLT3* or *NPM1* Mutation Status or Response to Chemotherapy Were Detected

**FIGURE 6:**
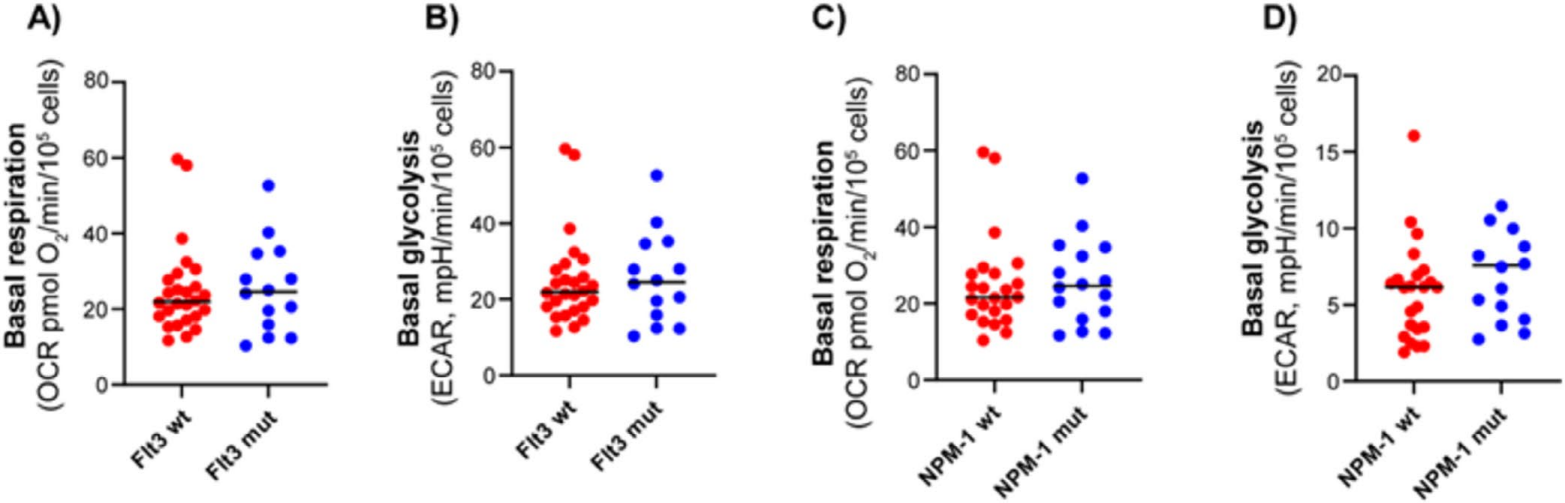
Relationship between the metabolic phenotype and *FLT3* and *NPM1* mutations. Comparison of mitochondrial respiration (oxygen consumption rate, OCR) and glycolysis (extracellular acidification rate, ECAR) between subsets with or without mutations in *FLT3* and *NPM1*. Basal rates of mitochondrial respiration and glycolysis are compared between groups with or without *FLT3* mutation (A, B) and *NPM1* mutation (C, D). All data are shown as mean of 4–8 technical replicates for each subject. Statistical difference was evaluated using Welch's test.

Molecular mutations are important drivers of leukemogenesis and have significant therapeutic and prognostic implications. We investigated whether there was a connection between cellular metabolic profiles and mutations in the two most commonly mutated genes in AML, *FLT3* and *NPM1*. However, when comparing subgroups of subjects with wild‐type and mutated forms of these genes, the rates of mitochondrial respiration and glycolysis were not significantly different (Figure 6). Hence, these mutations alone do not appear to be decisive for the metabolic phenotype of AML cells. Twenty‐four of the patients included in the study underwent standard AML induction treatment with cytarabine and an anthracycline (3 + 7 regimen) [12]. Of these, 20 patients achieved complete remission (CR) after the first induction. We compared the metabolic phenotype of patients who went into remission with those who did not, but no clear associations were detected. The same findings were observed when assessing overall survival (OS) in the patient cohort.

### 3.6 Glycolysis and Mitochondrial Respiration Correlate With Glucose Oxidation Rates Although Not With Glutamine Oxidation Rates

**FIGURE 7:**
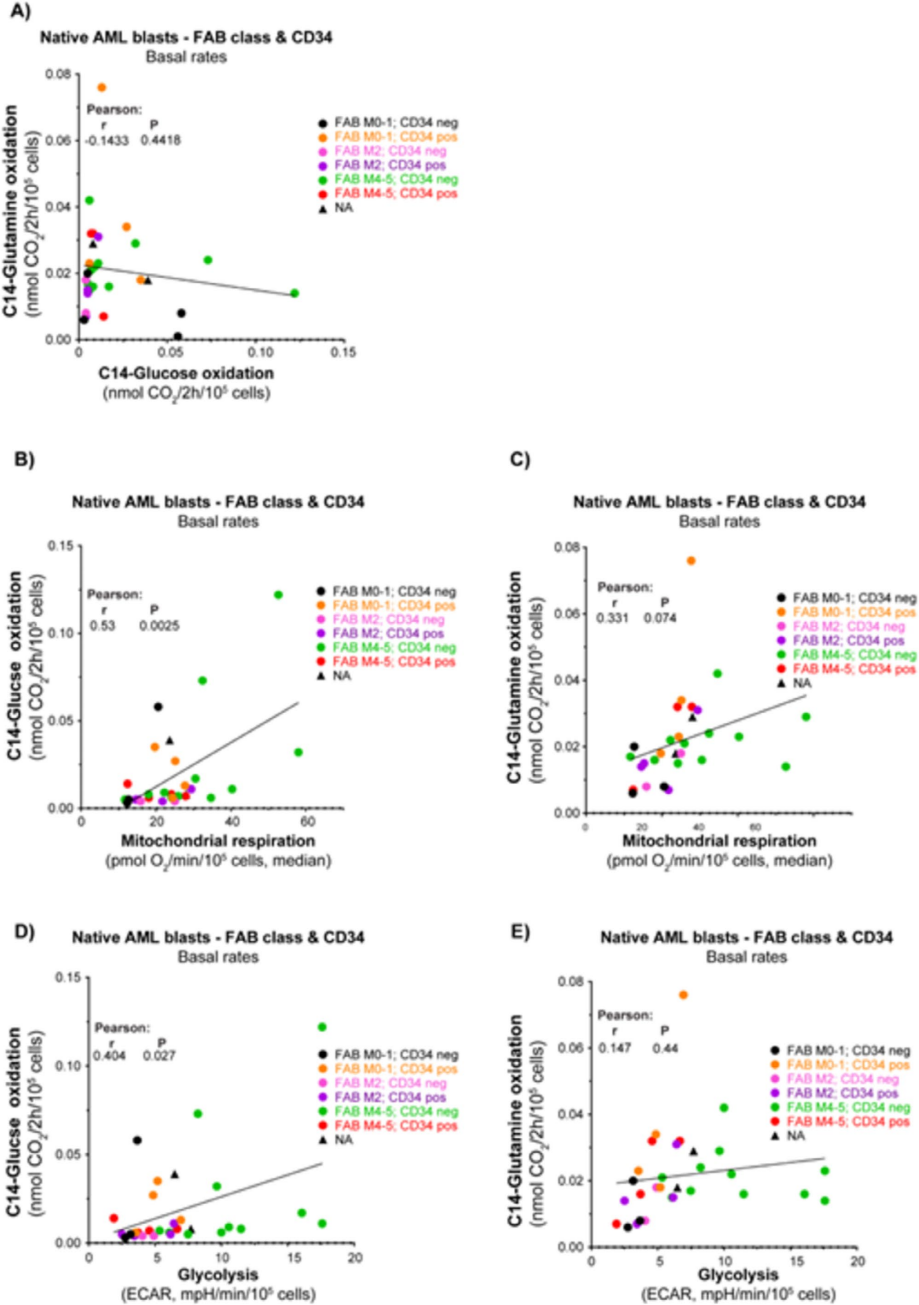
Glucose and glutamine oxidation rates. Glucose and glutamine oxidation rates were assessed by measuring the cellular production of ^14^CO_2_ from ^14^C‐labeled glucose/glutamine (nmol CO_2_/2 h/10^5^ cells), using primary AML samples (*n* = 31). (A) The relationship between the measured rates of glucose and glutamine oxidation. The relationship between mitochondrial respiration and (B) glucose oxidation rate and (C) glutamine oxidation rate. The relationship between glycolysis and (D) glucose oxidation rate and (E) glutamine oxidation rate. Data are shown as the mean of 5 technical replicates. Pearson correlation analysis and simple linear regression analysis were used.

To investigate possible differences in energy fueling, we provided ^14^C‐labeled glucose and glutamine (separately) to the cell cultures and quantified the oxidation product ^14^C‐CO_2_ released from the cultures. We obtained data for both substrates from 31 AML subjects. Our analysis revealed no clear association between glucose and glutamine oxidation rates (Figure 7A). For the entire cohort, there was a significant association between glucose oxidation rate and the rates of both glycolysis and mitochondrial respiration (Figure 7B,C). There was a trending association (*p* = 0.0737) between glutamine oxidation rate and mitochondrial respiration, but not versus glycolysis (Figure 7D,E). Notably, there was greater variation in glucose oxidation rates compared to glutamine oxidation, with certain patients exhibiting particularly distinct profiles.

## 4 Discussion

Our analysis of primary AML cells from 46 patients revealed significant variability in metabolic activity, highlighting the diverse metabolic phenotypes within this disease. Measurable rates of mitochondrial respiration and glycolysis were detected in 87% of the samples. The obtained metabolic profiles were further emphasized by the responses to OXPHOS inhibition by oligomycin, and by possible relationships with cell maturation, diagnostic markers, and adverse cytogenetics.

The functional analysis of primary AML cell metabolism shows important differences between patients and possible associations with maturation (FAB class), CD34 status, and adverse cytogenetics, consistent with previous studies [8]. Both under basal and OXPHOS‐inhibited conditions, the absolute rates of mitochondrial respiration and glycolysis showed a strong correlation, suggesting that they co‐vary depending on the overall metabolic state. However, the impact of oligomycin on mitochondrial respiration and glycolysis varied significantly among samples, with reductions in respiration ranging from 19% to 88% and increases in glycolysis ranging from 0% to 109%. A small change in these parameters after adding oligomycin strongly suggests that the cells are already in a state of anaerobic metabolism, whereas a large change may indicate a higher level of metabolic flexibility. Furthermore, the absolute respiratory rate may play a role, as it has been suggested that AML cells have increased mitochondrial mass without a corresponding increase in the functional capacity of the respiratory chain enzymes [13]. Such effects may lower the threshold for inducing metabolic stress mechanisms, which could represent a target for therapeutic intervention.

AML cells classified as FAB M4/M5 exhibited higher rates of both mitochondrial respiration and glycolysis compared to more immature phenotypes. Different metabolic characteristics in these subtypes are supported by other studies [8, 14, 15, 16]. These findings suggest that AML cells with more mature phenotypes have distinct metabolic profiles that may reflect their enhanced energetic requirements or metabolic adaptations. Furthermore, higher proliferation rates in these AML subtypes have been recognized for some time [17], suggesting that their elevated metabolic activity is likely linked to both their maturation signature and increased proliferative capacity. However, in our present study, no clear association to 7‐days proliferation rates and OCR and ECAR were detected. When both FAB classification and CD34 status were considered, the combination of M4/M5 classification and CD34 negativity was associated with significantly higher metabolic rates, aligning with a more pronounced glycolytic phenotype. Interestingly, the novel AML therapeutic, the BCL‐2 inhibitor, venetoclax, has shown clear benefits in treating AML [18]. However, it appears to be less effective in patients with the AML FAB M4/5 subtypes [16, 19]. This reduced efficacy is believed to be at least partly due to the distinct metabolic characteristics associated with the M4/5 subtypes of AML [20]. Specifically, these subtypes may rely more heavily on alternative metabolic pathways that circumvent the effects of venetoclax. The mechanisms underlying the more resistant venetoclax phenotype in the FAB M4/M5 cohort are not yet fully understood but likely involve several factors, including distinct mutation profiles such as *RAS* mutations [21], the presence of leukemia subpopulations known for their resistance to treatment [16], and bone marrow microenvironment factors, particularly the presence of AML‐associated macrophages (AAMs) [22]. Additionally, the unique metabolic properties of AML FAB M4/M5 could also influence the cellular response to therapy [23, 24, 25]. These underlying mechanisms require further exploration in larger studies, including investigations into whether changes in the metabolic environment may reduce reliance on BCL‐2 or alter the sensitivity of these cells to the drug. Finally, ex vivo drug‐response and multiomics profiling data, including metabolic phenotypes, could represent an attractive clinical translational approach for advancing AML treatment [26].

Our study highlights the metabolic differences associated with AML FAB M4/5 and the potential for pharmacologically targeting these patients in a more tailored manner. However, it is important to note that the FAB M4/5 patient cohort likely contains cells from various subtypes, including AAMs [22]. Additionally, the bulk AML cell population consists of multiple subpopulations, with markers such as CD34 showing variability within these subsets [27]. A small subset of cells may also exhibit a leukemia stem cell (LSCs) phenotype [28]. Previous reports have also demonstrated that different AML subsets display distinct metabolic properties [29]. Larger pre‐clinical and clinical studies are warranted to further elucidate the significance of these distinct metabolic phenotypes within the broader AML cell population.

Adverse cytogenetics was linked to higher glycolytic rates; however, not to mitochondrial respiration. In contrast, intermediate cytogenetics showed a strong association between mitochondrial respiration and glycolysis. These findings suggest that cytogenetic abnormalities may influence the metabolic phenotype, particularly glycolysis, which could have implications for prognosis and treatment strategies. On the other hand, no significant differences in metabolic rates were observed between AML cells with wild‐type versus mutated forms of *FLT3* or *NPM1*. However, previous studies have highlighted distinct metabolic properties, particularly in *FLT3*‐ITD‐mutated AML [23, 25]. Several factors could explain the lack of observed differences in metabolic profiles in *FLT3*‐mutated samples, such as variations in the patient cohort, sample preparation methods, or experimental conditions. Additionally, other signaling pathways or cellular contexts may influence the metabolic phenotype in ways that were not captured in our model. Finally, it should be emphasized that mutations other than *FLT3* and *NPM1* are also important in the classification and prognostication of AML [4, 12, 30, 31]. These mutation profiles, along with their potential impact on metabolic phenotypes, should also be explored in future studies.

Several reports have concluded that a hypermetabolic phenotype with increased rates of mitochondrial respiration is associated with proliferation and growth in AML cells [32]. Our study suggests that the maturation state should also be considered in this context. The aerobic glycolysis phenotype, that is, the Warburg effect, is known to be associated with a proliferative state and may provide adaptation to hypoxia and/or mitochondrial dysfunction [6, 33, 34]. In our study, some samples showed a higher glycolytic rate relative to mitochondrial respiration. Experiments using specific laboratory in vitro and in vivo models of leukemia may not always capture the full spectrum of metabolic phenotypes observed in this study. In this study, we tried to measure the most native metabolic state possible in blood samples. It should also be emphasized that differences between leukemia cells derived from bone marrow and those derived from blood, as used in our study, can vary [35]. Blood samples are more likely to contain a higher proportion of mature, differentiated AML cells, which may not fully represent the behavior of leukemia stem cells or early‐stage blasts [22]. Additionally, blood samples are less likely to recapitulate the microenvironmental interactions seen in the bone marrow. Nonetheless, we believe that our study of blood‐derived leukemia cells provides valuable insights into potential outcomes following treatment with metabolic stressors. Finally, the potential impact of cryopreservation on the metabolic rates of AML blasts cannot be ruled out, particularly with the methods used in our study [36].

Specific mechanisms that may underline or adhere to the observed metabolic profiles include sensitivity to apoptosis caused by metabolic stress, such as inhibition of mitochondrial fatty acid oxidation (etomoxir) [37]. Recently, it was suggested that AML cells upregulate SLC25A51 to decouple the mitochondrial NAD+/NADH ratio, providing a proliferative [38, 39] advantage by supporting oxidative reactions from various fuels [40]. Furthermore, leukemic mitochondria are poised to consume ATP, and OXPHOS repression supports aggressive disease dissemination in AML [32]. Increased matrix ATP consumption through the reverse ATP synthase reaction, to polarize the inner membrane, may play a role as a protection mechanism involved in chemotherapy resistance. Our screening of mitochondrial respiration and glycolysis activities in cells from 46 AML patients supports that various aspects of these mechanisms may be relevant. Elucidation of these differences could possibly lead to targeted clinical approaches in specific patient subsets. AML exhibits distinct metabolic dependencies, making targeted therapies an emerging approach [6, 29, 41]. Potential strategies include inhibiting OXPHOS with drugs like venetoclax. Other approaches include targeting glutamine metabolism using glutaminase inhibitors to disrupt amino acid utilization critical for AML survival [42]. Alternatively, lipid metabolism inhibitors, such as fatty acid oxidation blockers, show promise in starving AML cells of essential energy sources [43].

The cell samples of our cohort are cryopreserved, and these can be thawed and cultured for several days to study physiological aspects and molecular characteristics such as proliferation, cytokine responses, and secretion [35]. During cell culture, proliferative subpopulations are expected to have a selection advantage and become dominant after some time. In the present study, we aimed to investigate the native composition of cells and therefore performed the analyses with minimal time under ex vivo conditions. Apoptosis following thawing could have influenced the metabolic data; although we performed the Seahorse assays as quickly as possible after thawing, the presence of apoptosis may still have affected the results. Performing assays on fresh samples could, in some cases, offer an advantage; however, logistical constraints often limit this approach. The viability was generally high, which facilitated the characterization of vital metabolic properties. We observed that loss of viability sometimes occurred during the course of the measurement, which may have influenced the metabolic activity and data for some samples. Samples showing no meaningful metabolic activity were excluded from the statistical analysis, but otherwise, all data were included to explore possible biological relationships. The experiments were performed blinded to facilitate unbiased interpretations. The data obtained are considered robust and reproducible, as we were able to identify and exclude particularly vulnerable descriptors, such as CCCP‐uncoupled respiration. Notably, cell lines are adapted to cell culture conditions and show higher metabolic rates and more tolerance to experimental manipulations compared to primary cells. Additionally, growth and proliferation rates in primary cells are not directly comparable to results obtained from cell lines [44]. Finally, it is important to emphasize that studying primary blood versus bone marrow leukemic cells in AML in vitro can provide distinct insights due to differences in cell composition and microenvironmental influences. Bone marrow samples may better reflect the disease niche, which could also impact metabolic characteristics [8]. Future studies comparing the metabolism of primary blood and bone marrow leukemic cells should be conducted to deepen our understanding of these differences.

In summary, our analysis of primary AML cells from 46 patients revealed significant variability in metabolic activity, highlighting diverse metabolic phenotypes within the disease. Notably, AML cells classified as FAB M4/M5 exhibited higher metabolic rates compared to less mature phenotypes, suggesting that maturation state and cytogenetic abnormalities influence metabolic characteristics. These findings underscore the potential for targeted therapeutic strategies based on specific metabolic profiles, particularly for AML patients with M4/5 subtypes who may require different treatment approaches due to their distinct metabolic adaptations.

## Author Contributions

**Sissel Dyrstad:** formal analysis (lead), investigation (lead), methodology (lead). **Kimberley Joanne Hatfield:** conceptualization (supporting), investigation (supporting), project administration (supporting), supervision (supporting), writing – original draft (supporting), writing – review and editing (supporting). **Endre Stigen:** data curation (supporting), formal analysis (supporting), investigation (supporting), methodology (lead), visualization (supporting). **Marte Karen Brattås:** methodology (equal). **Karl Johan Tronstad:** conceptualization (lead), funding acquisition (supporting), supervision (lead), visualization (supporting), writing – original draft (lead), writing – review and editing (supporting). **Håkon Reikvam:** conceptualization (supporting), funding acquisition (lead), project administration (lead), resources (supporting), supervision (supporting), writing – original draft (lead), writing – review and editing (lead).

## Ethics Statement

The collection and use of samples for this study were approved by the Regional committees for medical and health research ethics (REK), both for biobanking and in vitro experimental research (REK 1750/2015 and REK 480847/2022). Registration of collected samples is also approved by the Norwegian Data Protection Authority (reference 02/1118‐5).

## Conflicts of Interest

H.R. has consulted Novartis and Glaxo Smith Kline. The other authors declare no conflicts of interest.

## Supporting information


Data S1.

## Data Availability

Data from the study are available upon reasonable request to corresponding authors.
